# Management of Traumatic Spigelian Hernia: A Case Report and Literature Review

**DOI:** 10.7759/cureus.6213

**Published:** 2019-11-21

**Authors:** Allison M Yee, Seyed B Jazayeri, Olivia Mac, Sarkis Arabian, Michael Neeki

**Affiliations:** 1 Emergency Medicine, Arrowhead Regional Medical Center, Colton, USA; 2 Surgery, Arrowhead Regional Medical Center, Colton, USA; 3 Internal Medicine, Arrowhead Regional Medical Center, Colton, USA

**Keywords:** spigelian hernia, traumatic, abdominal wall hernia

## Abstract

Traumatic abdominal wall hernias comprise less than 1% of all abdominal wall hernias. We present a 22-year-old male sustaining a traumatic Spigelian hernia resulting from striking a guardrail while snowboarding. In addition, the patient was found to have injuries to the serosa of the small bowel and mesentery, which were repaired during emergent surgery. A hybrid surgical approach was used to repair the defect using both laparoscopy and an incision over the abdominal wall defect.

## Introduction

Traumatic abdominal wall hernias (TAWH) are uncommon and occur in less than 1% of all blunt abdominal traumas [1]. Despite more than 15,000 cases reported annually in some academic censuses, the literature lacks strong evidence for any standardized method of the management of TAWH. The first case was described in the literature by Selby in 1906 [2]. TAWH is defined as a new herniation due to blunt abdominal trauma with intact overlaying skin. Although a traumatic hernia can occur in any region of the abdomen, lateral and lower quadrant hernias are more common. A rare subtype of TAWH is a traumatic Spigelian hernia, which is diagnosed based on the location of the hernia at the semilunar line, lateral to the rectus abdominis muscle.

In reported cases, traumatic Spigelian hernias have been associated with injuries caused by a variety of mechanisms including handlebar injuries, falls from height, as well as motor vehicle collisions [3]. We present an unusual case of a 22-year-old male complaining of right lower quadrant abdominal pain after slamming into a guardrail while snowboarding at a regional ski resort.

## Case presentation

**Figure 1:**
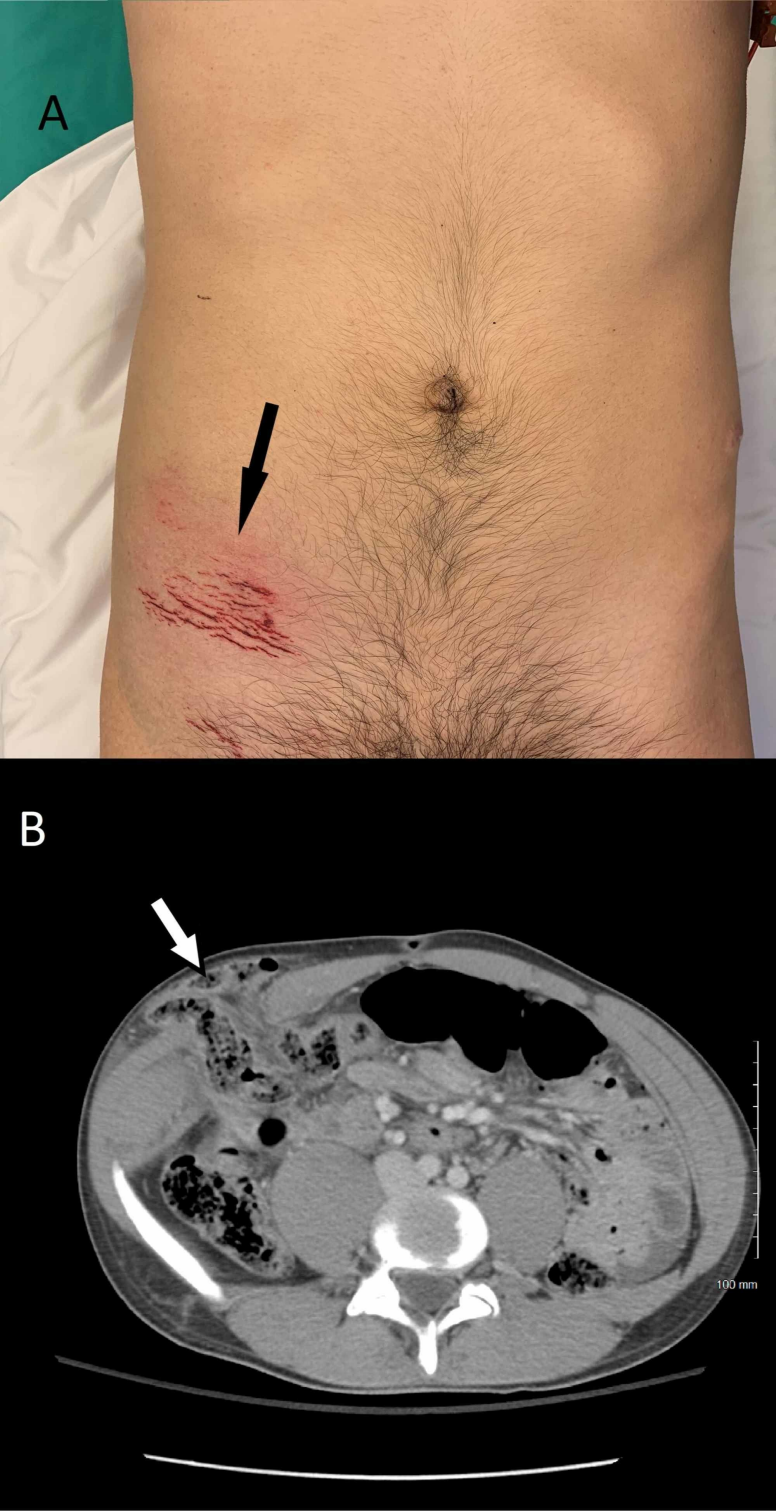
(A) Abrasion and hernia in the right lower quadrant of the abdomen while patient is supine indicated by the black arrow. (B) Contrast-enhanced computed tomography of the abdomen and pelvis which demonstrates a right-sided ventral hernia in the abdominal wall with its content of mesenteric fat and bowel loops indicated by the white arrow.

A 22-year-male was transported by emergency medical services from an area ski resort to a regional trauma center. The patient while snowboarding lost control and slammed into the guardrail. The patient complained of severe abdominal pain at the scene and was reported to have an enlarging hematoma in the right lower quadrant of the abdomen. The patient continued to have severe abdominal pain upon presentation to the trauma center. His vital signs on arrival to Arrowhead Regional Medical Center included a blood pressure of 111/87, pulse of 104, temperature of 98.1°F, and respiratory rate of 18 with an oxygen saturation of 99% on room air. On the secondary trauma assessment, a 10 cm by 5 cm bulge over the right lower quadrant of the abdomen accompanied by skin ecchymosis and abrasions was noted (Figure 1A). In addition, the patient sustained a 1.5 cm laceration of the right lower side of his scrotum.

The patient had a remote history of attention deficit hyperactivity disorder; however, he was not taking any medication for this condition. During the initial evaluation, he admitted to history of polysubstance abuse on the night prior to the traumatic incident. The urine drug screen confirmed his history of drug use disorder indicating the presence of cocaine, marijuana, and methamphetamine metabolites in his urine. The complete blood cell count demonstrated a leukocytosis of 15.1 × 10^9^/L, a hemoglobin of 12.9 g/dl, a hematocrit of 38.1, and platelets of 265 × 10^9^/L. Basic metabolic panel was within normal limits except for a carbon dioxide of 23 mEq/L. Coagulation tests resulted in a prothrombin time of 14.6, international normalized ratio 1.2, and partial thromboplastin time of 22.3. Urinalysis showed trace blood with five to ten urine casts per low power field.

Bedside point-of-care ultrasound indicated possible bowel in the right lower abdomen, and the focused assessment with sonography for trauma was positive with trace free fluid in the pelvis. Computed tomography (CT) scan with intravenous contrast of the abdomen and pelvis revealed the presence of small bowel in the abdominal bulge and a defect in the arcuate line compatible with a traumatic Spigelian hernia (Figure 1B). The patient was informed about the condition and subsequently underwent emergent surgery for the management of the hernia and further assessment of possible injuries to his intra-abdominal organs.

**Figure 2:**
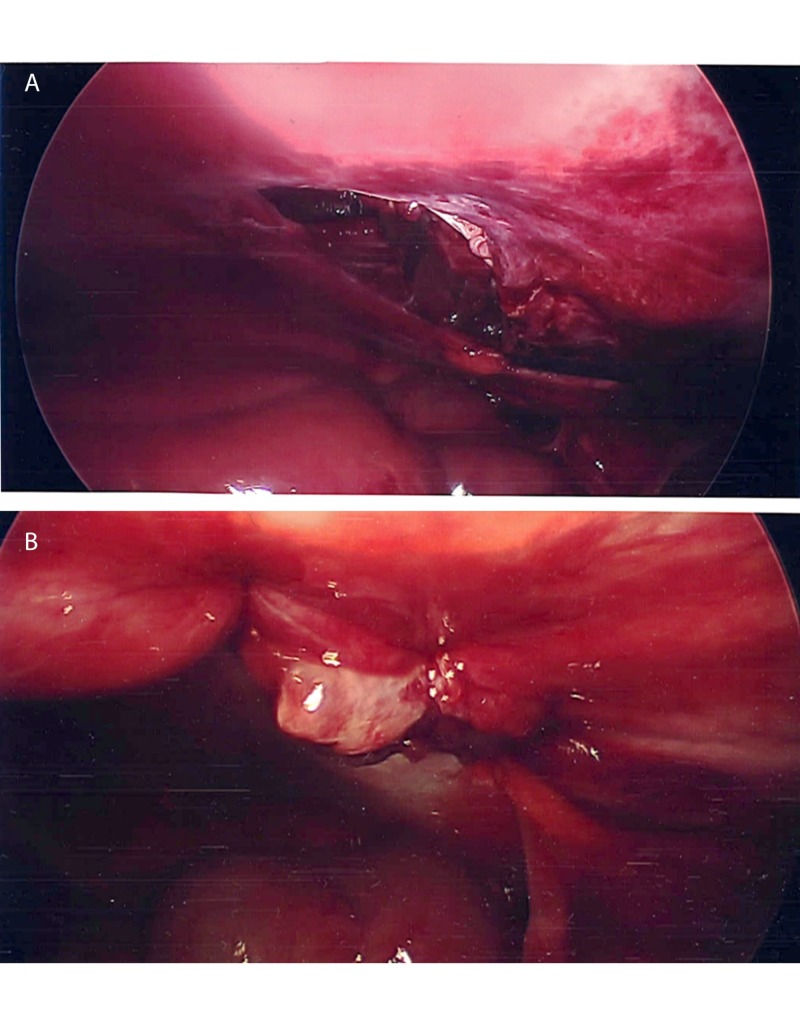
Laparoscopic views of abdominal wall defect (A) before closure and (B) after closure with interrupted sutures each indicated by an arrow.

While the patient was undergoing induction of anesthesia, after administration of the paralytic agent, the hernia self-reduced. An incision was made over the skin overlying the hernia and upon dissection through the subcutaneous layer, small bowel was visualized. Through the same incision, a 12 mm trocar was introduced into the abdomen, for laparoscopic insufflation of the peritoneal cavity to further evaluate for other major organ injuries. In addition, a 5 mm incision was made after insufflation in the left upper quadrant of the abdomen and a 30^o^ laparoscope (Olympus, WA50373B) was introduced to evaluate the hernia defect (Figure 2A). The small bowel was lifted through the abdominal wall defect and examined for possible injuries. A 1 cm small-bowel rupture was found and primarily repaired with simple sutures. Three additional mesenteric tears were also detected and were primarily repaired using simple interrupted sutures.

After evaluating the small bowel for possible injuries, the abdomen was irrigated with normal saline and the fascial defect was primarily closed with simple interrupted sutures. The external oblique and transversalis muscles were used to perform a tissue repair with multiple interrupted sutures (Figure 2B). At the end of the tissue repair, a relaxing incision was made in the external oblique muscle and the skin was loosely closed with interrupted subcutaneous sutures. The patient was then admitted to the trauma service for further observation and was discharged on postoperative day 4 without any complications with outpatient follow-up.

## Discussion

Traumatic Spigelian hernias are a rare type of hernia that account for less than 1% of all abdominal wall hernias [4-6]. Blunt abdominal trauma causes an abrupt increase in the intra-abdominal pressure leading to herniation through anatomically weak points in the fascia. In the case of Spigelian hernias, the weak point resides lateral to the rectus abdominis. Table 1 summarizes the cases of traumatic Spigelian hernias and their elected surgical management techniques [7-16]. Interestingly, all reported cases were in males, with 70% of reported cases in the pediatric population. This pattern of cases with a male predominance is also reported in other traumatic injuries but is not clearly explained [3,17].

The management of TAWH has been evolving overtime. In a patient presenting with an abdominal hernia after blunt trauma, the recommended diagnostic modality of choice is CT of the abdomen and pelvis with intravenous water-soluble contrast [18]. Although ultrasound can be helpful in the diagnosis, it does not provide detailed information about the hernia or other possible concurrent injuries in the abdomen and pelvis [18]. CT images provide valuable information about the content of the hernia, size of the defect, and other injuries in the abdomen and pelvis [7,15,18].

Traditionally, a midline laparotomy with repair of the hernia and exploration of the abdomen and pelvis has been recommended. However, recent studies have shown that the incidence of injuries that require surgical repair in patients with TAWH varies between 31% and 56% [17]. Moreover, the need for urgent repair of the hernia itself is questionable. The outcome of recent studies indicates that urgent hernia repairs have a higher recurrence rate [19]. A retrospective study of 80 patients with TAWH revealed that 71% of patients with TAWH were managed non-operatively, with no reported sequelae or symptoms on follow-up visits [17]. It is important to note that the follow-up for the reported cases was limited to only three years after the initial incident, and no long-term outcome was studied [17].

In another report of 34 TAWH cases, the author revealed that 50% of patients required surgery for associated intra-abdominal injuries [19]. Bowel and mesenteric injury were the most common concurrent injuries in TAWH requiring emergent surgical repair [19]. In addition, Netto et al. reported that 44% of patients undergoing operative management had bowel injury necessitating resection [19]. In this case, we discovered a contained bowel rupture with multiple mesenteric tears requiring surgical repair leading to the primary repair of the injuries and no resection was required.

The optimal method of hernia repair, including the use of mesh in repair of the TAWH, is debatable. The two most common methods of repairing traumatic and spontaneous Spigelian hernias are through exploratory laparotomy or incision of the abdominal wall over the defect (Table 1) [7-16]. Historically, exploratory laparotomy was preferred in identifying and repairing intra-abdominal injuries in cases of traumatic hernias [7].

**Table 1:** A summary of studies reported in the literature as traumatic Spigelian hernia and their respective repair type

Study	Age/gender	Etiology	Site of injury	Repair type
Aravinda et al. [7]	38 M	Fall from tree	Right lower abdomen with a gangrenous and perforated bowel	Exploratory laparotomy with temporary ileostomy placement
Landry [8]	14 M	Motorcycle handlebar	Left lower quadrant	Incision of abdominal wall defect with tissue repair
Herbert and Turner [9]	7 M	Bicycle handlebar	Left lower quadrant	Incision of abdominal wall defect with tissue repair
Mitchiner [10]	7 M	Bicycle handlebar	Left upper quadrant	Incision of abdominal wall defect with tissue repair
Mancel and Aslam [11]	7 M	Bicycle handlebar	Left lower quadrant	Incision of abdominal wall defect with tissue repair
Goliath et al. [12]	11 M	Bicycle handlebar	Right lower quadrant	Incision of abdominal wall defect with tissue repair
Lopez et al. [13]	14 M	Bicycle handlebar	Left lower abdomen	Laparoscopic with suture repair
Thakur et al. [14]	9 M	Bicycle handlebar	Right lower abdomen	Incision of abdominal wall defect with tissue repair
Gates et al. [15]	19 M	Fall off motorbike	Right lower abdomen with right colonic injury	Exploratory laparotomy, bowel resection with stapled anastomosis and suture repair of abdominal wall
Tinney et al. [16]	57 M	Fall from ladder	Left lower quadrant	Exploratory laparotomy

Using a midline incision provides a better exposure, opportunity to explore other organs, and a chance to close the defect from the inside. However, the impact and outcome of utilizing the midline incision technique compared to incision over the defect itself is not well understood [17].

In this case, we applied a hybrid approach of both laparoscopy and incision over the abdominal wall defect to identify injuries and repair the hernia. Although the use of synthetic and biologic mesh is reported in the acute settings, in this case, we elected to use a primary tissue repair method without the use of mesh [17].

TAWH should be suspected in patients with blunt abdominal trauma presenting with abdominal pain and an obvious abdominal wall bulge. A contrast-enhanced CT of the abdomen and pelvis can show the defect site and presence or absence of abdominal contents in the hernia sac. Although the abdominal bulge may be the most obvious finding on the physical exam and imaging, other more common and life-threatening injuries should not be overlooked [7,15]. A high-impact injury causing hernia formation can also lead to solid organ damage and consequent hemorrhagic events in the abdomen.

## Conclusions

Given the high number of cases reported in the recent decade, the management of TAWH is evolving. Whether all asymptomatic traumatic hernias should undergo surgical exploration to evaluate for intra-abdominal organ injury with emergent repair of the hernia remains unclear. However, in select patients with no other intra-abdominal injury, a non-operative management with or without delayed repair of the TAWH may be safe and feasible. A combination of open surgical repair of the hernia defect and laparoscopic examination of the abdominal and pelvic content provides an alternative approach to the traditional midline laparotomy.
